# Impact of Ultraviolet Radiation on Skin and Blood Melanin Traits in Xichou Black-Boned Chicken: A Transcriptomic and Metabolomic Study

**DOI:** 10.3390/ani15020141

**Published:** 2025-01-08

**Authors:** Xinlu Li, Zhongxiao Tian, Haojie Li, Lei Tan, Yong Zhang, Changrong Ge, Kun Wang

**Affiliations:** College of Animal Science and Technology, Yunnan Agricultural University, Kunming 650201, China; lixinlu1028@126.com (X.L.); 17863527659@163.com (Z.T.); hao18987269286@163.com (H.L.); tl622726@163.com (L.T.); zy10242025@163.com (Y.Z.)

**Keywords:** ultraviolet radiation, Xichou Black-boned Chicken, blackness, transcriptomics, metabolomics

## Abstract

This study investigated the effects of ultraviolet A (UVA) radiation on the blackness traits of Xichou Black-boned Chickens and their underlying molecular mechanisms through combined transcriptomic and metabolomic analyses. A total of 240 one-day-old Xichou Black-boned Chickens were randomly divided into four groups and exposed to different durations of UVA radiation at an intensity of 47 μW/cm^2^. Skin blackness was measured at 1, 22, and 45 days of age, and blood and pectoral skin samples were collected at 45 days for analysis. The results showed that an exposure time of 1 h per day at 47 μW/cm^2^ was ideal for enhancing the blackness traits of the chickens, while exposure times exceeding 3 h led to a significant increase in mortality. Omics studies revealed that the mTOR pathway and tryptophan metabolism pathway are potentially involved in the biological processes by which UVA radiation affects the blackness traits of Xichou Black-boned Chickens, with the *FZD3* gene being a candidate gene for these effects. These findings provide theoretical foundations and references for understanding the molecular regulation of blackness traits in these chickens and the potential application of UVA radiation in their production.

## Simple Summary

This study looked at how UVA light affects the dark color of a special kind of chicken called Xichou Black-boned Chicken. We wanted to see how different amounts of UVA light could change the chicken’s dark skin and understand why this happens at a molecular level. We found that a short daily exposure to UVA light can make the chickens darker, but too much light can be harmful. Our research suggests that certain pathways in the chicken’s body, like the mTOR pathway and tryptophan metabolism, play a role in this color change. We also identified a gene, *FZD3*, that might be important. This study helps us understand how to breed these chickens with the desired dark color and could be useful for chicken farmers and breeders.

## 1. Introduction

Solar radiation, categorized by wavelength into ultraviolet (UV) radiation, visible light, and infrared radiation, exerts complex and profound effects on living organisms [1]. Ultraviolet radiation, situated between violet light and X-rays, has a dual impact on animal health: excessive exposure can lead to skin pigmentation, photoaging, and increased cancer risk, and is closely linked to eye diseases such as cataracts [2,3,4]; however, moderate UV exposure is crucial for the synthesis of vitamin D and endorphins within the body [5]. In the poultry industry, empirical evidence has demonstrated that appropriate UV exposure can promote bone growth, enhance growth performance, and bolster bone strength in broilers [6,7,8,9]. Melanin, a multifaceted pigment present in various forms, including eumelanin, pheomelanin, neuromelanin, and pyomelanin [10,11], all derive from distinct biochemical pathways that involve the catalytic activity of tyrosinase and related TRP-1 and TRP-2 proteins [12,13].

In recent years, native chicken breeds have become a favorite on the dining tables of consumers in many Asian countries, including China. Compared to industrially farmed broilers, Chinese indigenous chickens are prized for their superior taste and nutritional value, carving out an important market niche [14,15]. In Yunnan, the Black-boned chicken stands out for its unique flavor, attributed to a higher content of umami peptides and aromatic compounds such as aldehydes, alcohols, and ketones in its meat [16,17], which has earned it a prominent place in the local poultry market.

The Xichou Black-boned chicken, primarily produced in Xichou County, Wenshan Prefecture, Yunnan Province, is also known as the Wenshan Mountain Black-boned chicken. This breed is a result of careful selection by the local people over a long period of agricultural development [18]. It is renowned for its robustness, disease resistance, black skin and bones, tender and flavorful meat, and overall high quality, making it a distinguished local variety that thrives on coarse feed [19].

Based on preliminary observations, there is a marked difference in the degree of blackness between caged and free-range Xichou Black-boned Chickens—the latter are directly exposed to natural UV radiation, whereas the former is solely exposed to artificial lighting. This observation led us to hypothesize that UV radiation may regulate melanin synthesis in Xichou Black-boned Chickens. Although UV irradiation is more commonly applied in plant growth research, its use in avian studies remains limited [20,21]. To address this gap in the literature, this study aims to elucidate the mechanism by which UV radiation influences the black traits of Xichou Black-boned Chickens, employing integrated transcriptomic and metabolomic analysis techniques. The objective is to identify differentially expressed genes and metabolites, thereby clarifying the molecular basis of black trait variations in Xichou Black-boned Chickens under UV radiation. This approach not only fills a knowledge gap in the field but also offers new insights into the scientific management of Xichou Black-boned Chicken farming and the application of UV radiation.

## 2. Materials and Methods

### 2.1. Experimental Animals and Grouping

This study employed 240 Xichou Black-boned Chickens, hatched from eggs supplied by the Xichou County Zhengyuan Agriculture Preservation Farm. These chicks were conceived through artificial insemination of healthy parent birds and hatched at the chicken farm affiliated with Yunnan Agricultural University. Upon hatching, the chicks were randomly allocated into four equal groups, comprising 60 chicks each, and were co-reared until they attained an age of 45 days. Throughout this period, standardized management practices were implemented, with the duration of UV radiation exposure being the sole variable.

The experimental design comprised four treatment groups: no UV radiation exposure (0 h), 1 h of daily UV exposure, 3 h of daily UV exposure, and 6 h of daily UV exposure. All groups were subjected to the same UV intensity of 47 μW/cm^2^, with the light source positioned 10 cm above the cages to ensure uniform irradiation. The UV irradiance was monitored using a Tamas Radiometer, and the experimental area was thoroughly enclosed with UV-resistant shading material to prevent external light interference and cross-contamination between groups.

### 2.2. Measurement of Growth Performance

To evaluate the effects of different UV irradiation durations on the growth performance and mortality of XiChou Black-bone chickens, body weight was measured at a fixed time before morning feeding each week starting from day 1 post-hatching. Thirty chicks were randomly selected from each group for weighing, and their weights were recorded to calculate the average weight and standard deviation. Daily health observations were conducted, and mortality rates were recorded. All weighing was performed using a calibrated electronic scale with a precision of 0.001 kg (measured in kilograms and displaying three decimal places) to ensure accuracy. During each weighing, the chickens were placed in the same position on the scale, which was kept level.

To assess the impact of UV exposure on mortality, a Chi-square test was conducted. The analysis compared mortality rates across different UV exposure groups to determine if there were significant differences.

### 2.3. Melanin Content Measurement

To investigate the effects of different UV exposure durations on skin darkness (melanin content) in Xichou Black-boned Chickens, this study began measuring melanin content from the first day post-hatching. Using a color difference meter (Konica Minolta Sensing, Inc., Osaka, Japan, Model CR-400) set to Lab mode, we recorded the L value (brightness indicator, ranging from 0 for complete black to 100 for complete white). A lower L value indicates higher melanin content and darker skin, while a higher L value indicates lower melanin content and lighter skin.

At 22 and 45 days of age, 16 male and 16 female chickens from each group were selected to measure the L value of their left chest and leg skin at a fixed time before morning feeding. Each measurement was taken three times to ensure data reliability. The L values were averaged for each chicken using Excel, and standard deviations were calculated. The final results are presented as mean ± standard deviation (mean ± SD).

### 2.4. Determination of Melanin Content in Blood

At 45 days of age, 3 mL of blood was collected by venipuncture from the wing vein (brachial vein), and the melanin content in the blood was directly measured using the ELISA method. The Chicken Melanin ELISA Rapid Test Kit, manufactured by Shanghai Xuanze Kang Biotechnology Co., Ltd., Shanghai, China, was employed for this purpose. The blood samples were combined with microplates pre-coated with melanin antibodies, followed by the addition of standard substances and HRP-labeled detection antibodies. After incubation and washing, TMB was used as the substrate for color development. Under the catalysis of peroxidase, the solution changed from colorless to blue and then to yellow upon acidification, with the depth of color being directly proportional to the concentration of melanin. The absorbance (OD value) of the samples was measured at a wavelength of 450 nm using a Thermo Scientific Multiskan FC Microplate Spectrophotometer, Waltham, MA, USA, Model 3001. A concentration-OD value linear regression curve was plotted from the standard substance using Excel software to accurately calculate the concentration of melanin in the blood samples.

### 2.5. Slaughter and Tissue Sample Collection

At 45 days of age, 12 chickens (six males and six females) from the groups with the most significant difference in L values (the 1 h UV exposure group and the 0 h UV exposure group) were slaughtered. Immediately post-slaughter, pectoral skin tissue samples were collected and placed into sterile cryotubes, which were then rapidly frozen in liquid nitrogen and stored at −80 °C for subsequent transcriptomic and metabolomic analyses.

### 2.6. Extraction of Total RNA from Pectoral Skin Tissue and Transcriptome Sequencing

Total RNA was extracted from the pectoral skin tissues of the chickens using TRIzol Reagent (Life Technologies, Carlsbad, CA, USA) following the manufacturer’s instructions. RNA concentration and purity were quantified using a NanoDrop 2000 spectrophotometer (Thermo Fisher Scientific, Wilmington, DE, USA). The integrity of the RNA was precisely assessed using the Agilent Bioanalyzer 2100 system (Agilent Technologies, Santa Clara, CA, USA) with the RNA Nano 6000 kit. After sample quality control, libraries were constructed, and following library quality control, paired-end sequencing (PE150) was performed on the Illumina NovaSeq 6000 platform by Kunming GenScript Biotech Co., Ltd., Kunming, Yunnan, China.

### 2.7. Validation of Target Gene Expression by RT-qPCR

Chicken (*Gallus gallus*) gene sequences were obtained from GenBank, and primers were designed using Primer 5.0 software and synthesized by Kunming GenScript Biotech Co., Ltd., Kunming, Yunnan, China. The primer sequences and annealing temperatures are listed in Table 1. Total RNA was extracted from the pectoral skin tissue of Xichou Black-boned Chickens using a Takara Bio Inc., Kusatsu, Shiga, Japan. Twelve samples were collected from each group (six males and six females). Following RNA quality control, reverse transcription was performed, and real-time quantitative PCR (RT-qPCR) was conducted using SYBR Green reagents to detect gene expression levels. Data were organized using Excel and analyzed for significance using SPSS 27.0. Gene expression levels were calculated using the 2^−ΔΔCT^ method, where ΔCT is the difference between the CT value of the target gene and the CT value of the reference gene, and ΔΔCT is the difference between the ΔCT of the target gene and the ΔCT of the reference gene.

### 2.8. Metabolite Sample Processing and LC-MS Analysis

Weigh 50 mg of the sample and mix it with 1000 μL of an extraction solution composed of methanol-acetonitrile-water (2:2:1) containing 20 mg/L of internal standard. After vortexing for 30 s, grind at 45 Hz for 10 min, then sonicate in an ice-water bath for 10 min. Subsequently, transfer the supernatant to an injection vial for analysis.

The LC/MS analysis is conducted using a Waters Acquity I-Class PLUS ultra-high-performance liquid chromatography (UPLC) system coupled to a Xevo G2-XS QTOF mass spectrometer (Waters Corporation, Milford, MA, USA), equipped with an Acquity UPLC HSS T3 column (Waters Corporation, Milford, MA, USA) (1.8 μm, 2.1 × 100 mm). In both positive and negative ion modes, the mobile phases A and B consist of 0.1% formic acid in water and 0.1% formic acid in acetonitrile, respectively, with an injection volume of 1 μL. The ESI source settings are as follows: positive ion mode voltage 2500 V, negative ion mode voltage −2000 V, cone voltage 30 V, ion source temperature 100 °C, desolvation gas temperature 500 °C, desolvation gas flow rate 50 L/h, and cone gas flow rate 800 L/h, with a mass-to-charge (*m*/*z*) range of 50–1200 for data acquisition.

### 2.9. Transcriptomic and Metabolomic Data Analysis and Integrated Analysis

#### 2.9.1. Transcriptomic Data Analysis

Effective sequence reads were aligned to the reference genome using an alignment tool (e.g., HISAT2 or STAR). Differential expression analysis between groups was conducted using DESeq2 [22], with screening criteria set at an adjusted *p*-value < 0.01 and a fold change ≥ 2 to identify differentially expressed genes (DEGs).

Gene Ontology (GO) enrichment and Kyoto Encyclopedia of Genes and Genomes (KEGG) pathway analyses were performed to uncover the functional and pathway enrichment of these DEGs. Particular attention was given to pathways related to melanocyte function and fatty acid metabolism, as these processes are closely associated with melanin synthesis. KEGG pathway enrichment was assessed using the hypergeometric distribution test to identify significantly enriched pathways.

Candidate genes involved in melanin formation under ultraviolet radiation were selected based on their differential expression patterns observed in the RNA-seq data and their involvement in melanin-related pathways. Genes that exhibited significant differential expression (adjusted *p*-value < 0.01 and fold change ≥ 2) and were part of pathways relevant to melanin synthesis, such as those involved in melanocyte function and fatty acid metabolism, were prioritized. This approach ensured that the selected genes were both differentially expressed and functionally relevant to the biological processes of interest.

#### 2.9.2. Metabolomic Data Analysis

Metabolomic data were acquired using MassLynx V4.2 and processed with Progenesis QI software version 2.5, including peak extraction and alignment. Compounds were identified through online databases (such as METLIN) and public databases, as well as a custom-built database, with theoretical fragment identification. The mass deviation thresholds were set at 100 ppm for precursor ions and 50 ppm for product ions. Normalized peak area information was subjected to principal component analysis (PCA) and Spearman correlation analysis to evaluate sample reproducibility. Compound classification and pathway information were obtained from KEGG, Human Metabolome Database (HMDB), and LipidMaps databases.

Fold changes and *t*-test *p*-values were calculated based on the groupings. Orthogonal projections to latent structures-discriminant analysis (OPLS-DA) models were built using the ropls package, and model robustness was validated with 200 permutations. Variable importance in the projection (VIP) values were computed. Differential metabolites were screened based on OPLS-DA model results, with criteria set at fold change (FC) > 1, *p*-value < 0.05, and VIP > 1 [23]. KEGG pathway enrichment analysis was conducted using the hypergeometric distribution test to assess the significance of differential metabolites. Pearson correlation analysis was used to calculate the correlation coefficients (CCs) between significantly differentially expressed genes and significantly differentially expressed metabolites.

#### 2.9.3. Integrated Analysis

Weighted Gene Co-expression Network Analysis (WGCNA) was performed on both transcriptomic and metabolomic datasets to reduce dimensionality. Genes and metabolites were clustered into distinct modules, with each module’s eigengene representing its overall expression pattern.

Correlations between the transcriptomic and metabolomic modules were calculated, and modules with at least one pair of inter-module correlations having a *p*-value < 0.05 were retained. Heatmaps were generated to visualize these correlations using color gradients, and hierarchical clustering was applied to group genes and metabolites with similar expression patterns. To identify common biological processes, shared pathways between the transcriptomic and metabolomic data were determined using a Venn diagram. A bubble plot was created to highlight KEGG pathways commonly enriched by both datasets.

## 3. Results

### 3.1. Growth Performance, Blood Melanin Content, and Melanin Degree of Xichou Black-Boned Chickens

#### 3.1.1. Effects of UV Exposure Duration on Growth Performance and Survival Rate

At 45 days of age, the effect of varying durations of UV radiation exposure on the body weight and survival rate of Xichou Black-boned Chickens was analyzed. The summarized data are presented in Table 2 and Table 3.

The results indicated that as the duration of UV exposure increased, the average body weight of chickens in each group tended to rise. Specifically, the mean body weight of the 6 h group (592.9 ± 74.6 g) was significantly higher than that of the 0 h group (556.8 ± 51.0 g) (*p* < 0.05). In contrast, there were no significant differences in mean body weight among the 0 h, 1 h, and 3 h groups (*p* > 0.05).

The Chi-square test revealed significant differences in mortality rates across the different UV exposure groups (χ^2^ = 10.672, *p* = 0.0136). The mortality rates were as follows: 0 h group (8.33%), 1 h group (8.33%), 3 h group (6.67%), and 6 h group (23.34%).

#### 3.1.2. Melanin Content in Blood at 45 Days of Age

The melanin content in the blood of Xichou Black-boned Chickens from different UV exposure groups was measured. As shown in Table 4, the order of melanin content from highest to lowest was: 1 h group > 3 h group > 6 h group > 0 h group (*p* > 0.05). Overall, it was found that the melanin content in the UV-irradiated group increased, but after the irradiation time was more than 1 h, the melanin content showed a trend of decreasing with the longer irradiation time.

#### 3.1.3. Measurement Results of Skin Hue (L Value) in Breast and Leg at 22 and 45 Days of Age

At 22 and 45 days of age, the skin hue (L value) of Xichou Black-boned Chickens from different UV exposure groups was measured for both the breast and leg skin. The results are presented in Table 5. At both ages, the L values of the breast and leg skin in the 1 h, 3 h, and 6 h groups were significantly lower than those in the 0 h group, with the 1 h group having the lowest L values. Overall, the longer the UV exposure time, the higher the L values of the skin, indicating that appropriate UV exposure time helps to enhance the skin hue of Xichou Black-boned Chickens.

Specifically, for the breast skin, at 22 days of age, the L values of the 1 h and 3 h groups were significantly lower than those of the 0 h and 6 h groups (*p* < 0.01), with no significant difference between the 1 h and 3 h groups or between the 6 h and 0 h groups (*p* > 0.05). At 45 days of age, there were significant differences between the 0 h, 1 h, 3 h, and 6 h groups (*p* < 0.01).

For the leg skin, at both 22 and 45 days of age, the L values of the 1 h, 3 h, and 6 h groups were significantly lower than those of the 0 h group (*p* < 0.01), and the differences between each group were also significant (*p* < 0.01).

During the period from 1 to 45 days of age, the L values of the breast and leg skin of Xichou Black-boned Chickens gradually increased, indicating that the skin hue became lighter over time. All experimental groups exhibited a consistent trend, reflecting the natural process of change in the skin hue of Xichou Black-boned Chickens.

A comprehensive analysis of the results indicates that the 1 h group of Xichou Black-boned Chickens had the lowest L values in both the breast and leg skin, along with the highest melanin content in the blood. This suggests that 1 h of daily UV exposure at 47 μW/cm^2^, without affecting the survival rate, can effectively enhance the skin hue characteristics of Xichou Black-boned Chickens.

### 3.2. Transcriptomic Results and Analysis

#### 3.2.1. Screening of Differentially Expressed Genes in the Chest Skin Tissue of Xichou Black-Boned Chickens

During the detection of differentially expressed genes, genes with a fold change (FC) ≥ 1 and a false discovery rate (FDR) < 0.05 were selected as the criteria. A total of 22 differentially expressed genes were screened between the 1 h group and the 0 h group in the chest skin tissue, including 5 upregulated genes and 17 downregulated genes.

**Figure 1:**
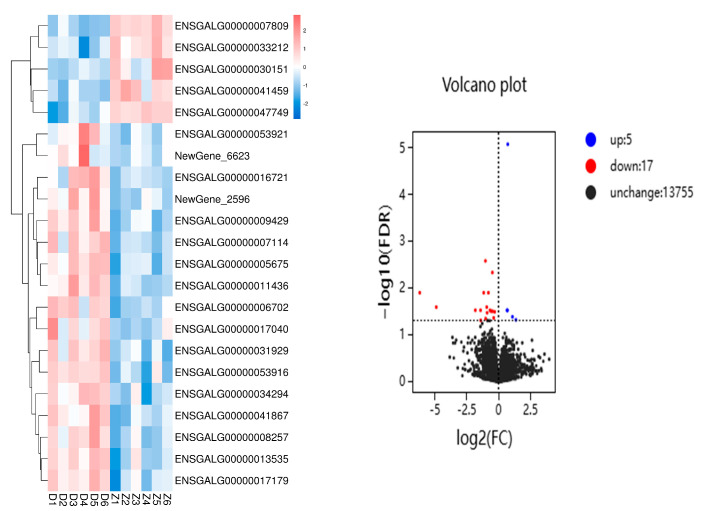
This figure presents the clustering analysis of differentially expressed genes between the 0 h and 1 h groups. The left panel shows a heatmap of gene expression clusters, with color gradients indicating the level of expression. The right panel is a volcano plot, where red circles represent upregulated genes and blue circles represent downregulated genes.

A clustering analysis was performed on the differentially expressed genes in the 0 h and 1 h groups. As can be seen from Figure 1, the 12 samples were clustered into two categories. The samples D1, D2, D3, D4, D5, and D6 from the 0 h group were clustered into one category, and the samples Z1, Z2, Z3, Z4, Z5, and Z6 from the 1 h group were clustered into another category. 

#### 3.2.2. GO Enrichment Analysis of Differentially Expressed Genes in the Chest Skin Tissue of Xichou Black-Boned Chickens

**Figure 2:**
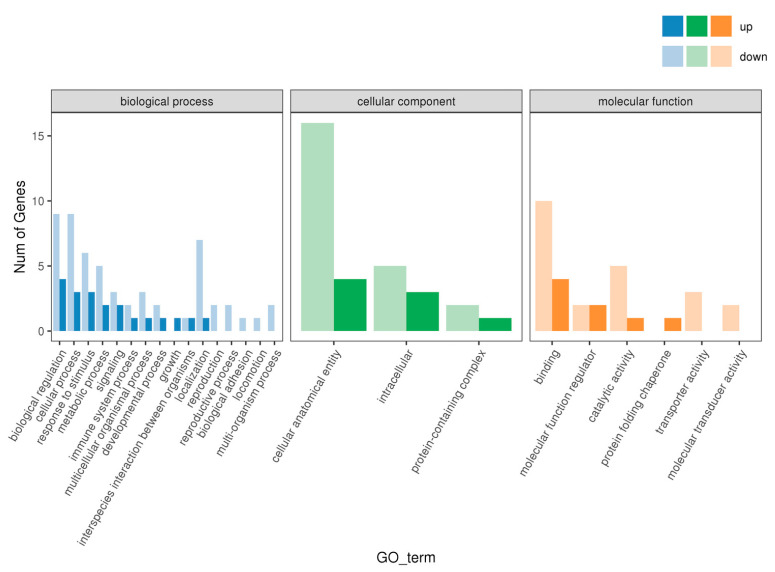
This figure presents the GO enrichment analysis results for differentially expressed genes between the 1 h and 0 h groups. The bar graph displays the number of genes enriched in various GO terms across three main categories: biological process, cellular component, and molecular function. Dark-colored bars indicate upregulated genes, while light-colored bars represent downregulated genes.

In this study, we conducted a GO enrichment analysis for the differentially expressed genes between the 1 h and 0 h groups. The results indicated that a total of 25 significant GO terms were enriched, as shown in Table 6 and Figure 2. In terms of biological processes, the genes were significantly enriched in key areas such as biological regulation, cellular processes, metabolic processes, signal transduction, and developmental processes. These pathways play central roles in the basic functions of cells and their response to the environment. In terms of molecular functions, the enriched GO terms mainly involved binding, catalytic activity, and transporter activity. Regarding cellular components, the genes were primarily enriched in cellular anatomical entities and intracellular regions.

#### 3.2.3. KEGG Enrichment Analysis of Differentially Expressed Genes in the Chest Skin Tissue of Xichou Black-Boned Chickens

**Figure 3:**
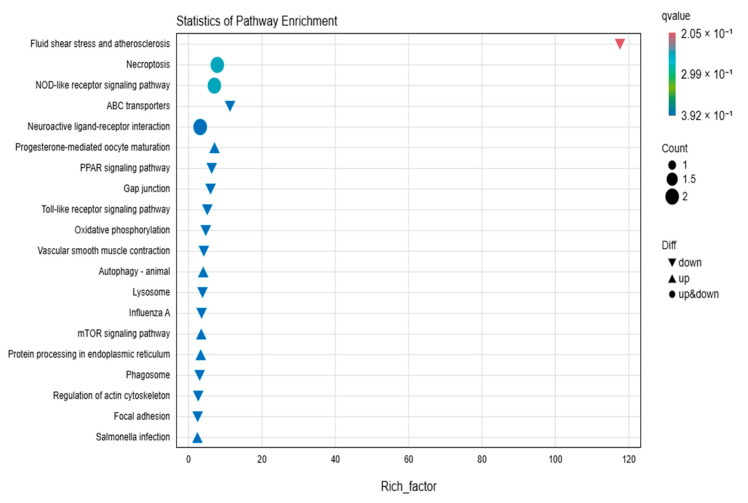
This figure presents the KEGG enrichment analysis of differentially expressed genes using a bubble plot. The bubble plot highlights the top 20 pathways with the smallest q-values. The size of the bubbles indicates the number of genes enriched in each pathway, and the color gradient reflects the q-value, with darker colors indicating lower q-values. Upregulated pathways are indicated by upward triangles, downregulated pathways by downward triangles, and pathways with both upregulated and downregulated genes by circles.

KEGG enrichment analysis of the 22 differentially expressed genes revealed the enrichment of 25 metabolic pathways (Figure 3). Notably, pathways related to melanocyte function and fatty acid metabolism, which are relevant to the melanin synthesis process, were significantly enriched. Specifically, the mTOR signaling pathway and the PPAR signaling pathway were identified as two significantly related signaling pathways. Several candidate genes involved in the melanin formation process of black-boned chickens under ultraviolet radiation were also identified, including *APOA1* (Apolipoprotein A-I), *FZD3* (Frizzled Class Receptor 3), *HSP90AA1* (Heat Shock Protein 90 Alpha Family Class A Member 1), *LPL* (Lipoprotein Lipase), and *RXFP1* (Relaxin).

#### 3.2.4. Validation of Candidate Gene Expression by Fluorescence Quantitative PCR

**Figure 4:**
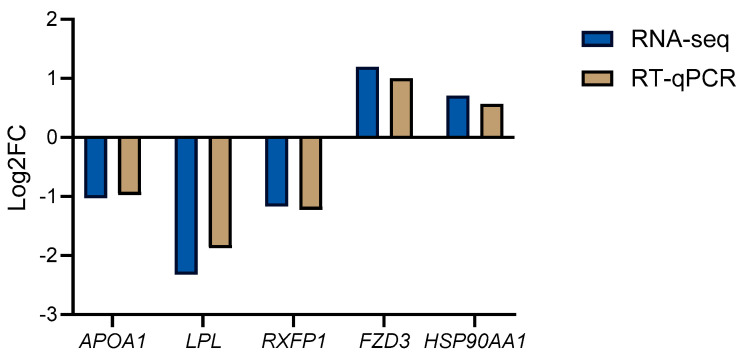
This figure compares the expression levels of candidate genes between RT-qPCR and RNA-Seq. The bar graph shows the log2 fold change (log2FC) for five genes: *APOA1*, *LPL*, *RXFP1*, *FZD3*, and *HSP90AA1*. Blue bars represent RNA-Seq results, while red bars represent RT-qPCR results. Upregulated genes are indicated by positive values and downregulated genes by negative values.

After calculating the fold change (FC) of the candidate gene expression in RT-qPCR, the values were transformed using the log2 function and compared with the log2FC values of the genes obtained from RNA-Seq. The results are shown in Figure 4, where the trends of up- and down-regulation of the aforementioned candidate genes are consistent between RT-qPCR and RNA-Seq methods.

### 3.3. Metabolomic Results

#### 3.3.1. Results of Differential Metabolite Screening

In the metabolomic analysis of chest skin tissue between the 1 h and 0 h groups, we detected a total of 5679 metabolites. By setting the criteria of VIP (Variable Importance in Projection) greater than 1 and a *p*-value less than 0.05, we identified 365 significantly differential metabolites between the two groups, with 186 up-regulated and 179 down-regulated.

**Figure 5:**
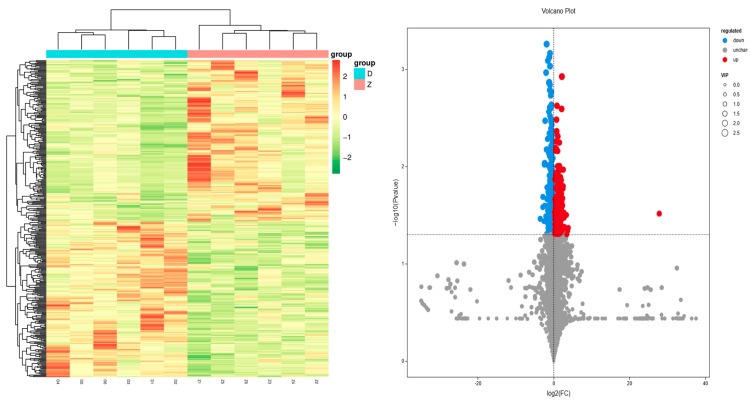
Clustering Heatmap and Volcano Plot of Differential Metabolite Expression. The left panel shows a heatmap of metabolite expression clusters, with color gradients indicating the level of expression. The right panel is a volcano plot, where red circles represent upregulated metabolites and blue circles represent downregulated metabolites. The size of the circles indicates the VIP (Variable Importance in Projection) values, with larger circles representing higher VIP values.

The clustering heat map and volcano plot of the differential metabolites are displayed in Figure 5. As shown in the figure, the expression patterns of metabolites among different samples exhibit a clear grouping trend, and there are significant differences in metabolites between the two groups.

The top 20 most abundant differential metabolites detected are listed in Table 7. The differential metabolites mainly include: carboxylic acids and their derivatives, fatty acyl groups, organic oxygen compounds, steroids and their derivatives, isoprenoid lipids, benzene and its derivatives, quinoline and its derivatives, flavonoids, glycerophospholipids, sphingomyelins, diazines, organic nitrogen compounds, coumarins and their derivatives, purine nucleotides, unsaturated hydrocarbons, macrolide lactones and similar compounds, pyrimidine nucleotides, benzofurans, benzoxazines, diphenylheptanes.

#### 3.3.2. Pathway Enrichment Analysis of Differential Metabolites

**Figure 6:**
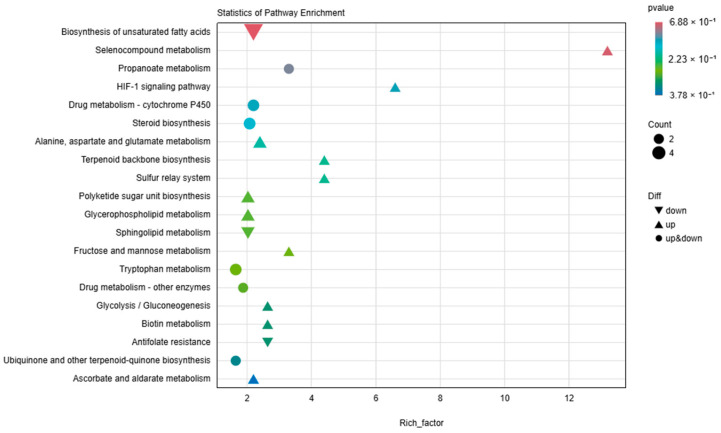
Pathway Enrichment Analysis of Differential Metabolites. This figure presents the KEGG enrichment analysis of differentially expressed metabolites using a bubble chart. The bubble chart highlights the top pathways with the smallest q-values. The size of the bubbles indicates the number of metabolites enriched in each pathway, and the color gradient reflects the q-value, with darker colors indicating lower q-values. Upregulated pathways are indicated by upward triangles, downregulated pathways by downward triangles, and pathways with both upregulated and downregulated metabolites by circles.

Through KEGG enrichment analysis, we identified the main metabolic pathways that differ between the UV-irradiated group and the normal light group (see Figure 6). These include amino acid metabolism, biosynthesis of secondary metabolites, pathways in cancer, carbohydrate metabolism, digestive system, lipid metabolism, metabolism of cofactors and vitamins, nervous system, nucleotide metabolism, signaling molecules, and interactions, as well as metabolism of xenobiotics.

After metabolic pathway analysis and literature research, we selected two metabolic pathways closely related to the synthesis of melanin and unsaturated fatty acids: tryptophan metabolism and biosynthesis of unsaturated fatty acids. In the tryptophan metabolism pathway, L-tyrosine was identified as a key differential metabolite; and in the biosynthesis pathway of unsaturated fatty acids, alpha-linolenic acid was also identified as an important differential metabolite.

### 3.4. Joint Analysis of Transcriptome and Metabolome

#### 3.4.1. Correlation Coefficient Matrix Heatmap and Hierarchical Clustering Heatmap

**Figure 7:**
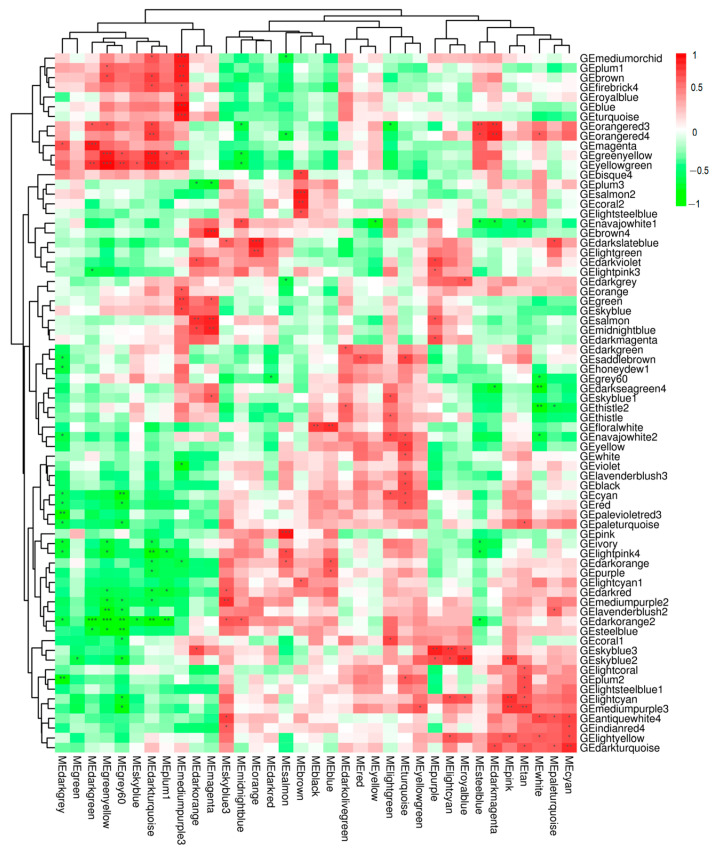
Correlation Coefficient Matrix Heatmap. This figure presents the correlation coefficient matrix heatmap for differentially expressed metabolites. The heatmap visualizes the pairwise correlations between metabolites, with color gradients indicating the strength and direction of the correlations. Red indicates positive correlations, while green indicates negative correlations. The intensity of the colors reflects the magnitude of the correlation coefficients. Asterisks indicate statistical significance levels: * represents *p*-value < 0.05, ** represents *p*-value < 0.01, and *** represents *p*-value < 0.001.

The correlation coefficient matrix heatmap (Figure 7) revealed significant correlations between differentially expressed genes and metabolites, with color gradients indicating the strength of inter-module correlations. Modules exhibiting *p*-values less than 0.05 were retained, highlighting strong associations between specific transcriptomic and metabolomic modules.

**Figure 8:**
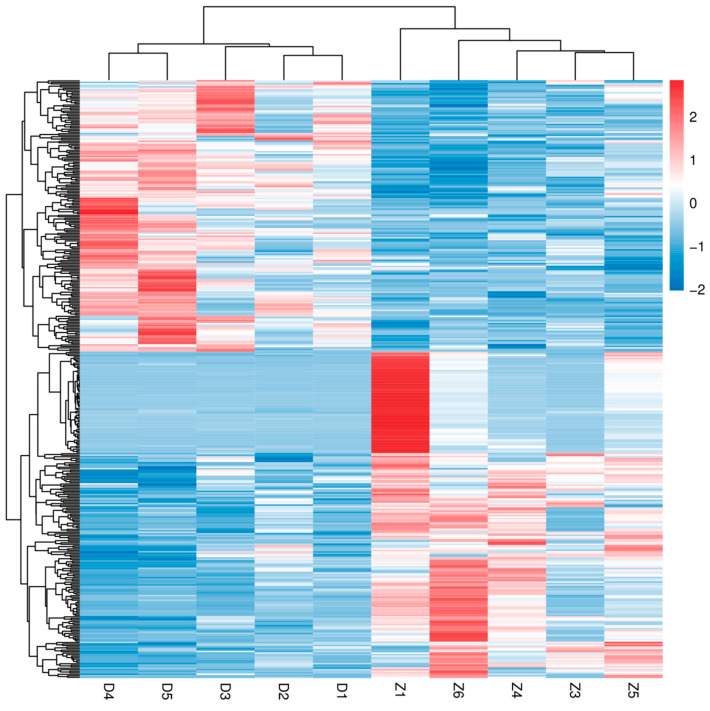
Hierarchical Clustering Heatmap. This figure presents the hierarchical clustering heatmap of differentially expressed genes. The heatmap visualizes the expression levels of genes across different samples, with color gradients indicating the level of expression. Red indicates higher expression levels, while blue indicates lower expression levels. The dendrograms on the left and top show the hierarchical clustering of genes and samples, respectively.

The hierarchical clustering heatmap (Figure 8) visually depicted the expression patterns of differentially expressed genes and metabolites. Genes and metabolites that clustered together showed similar expression profiles, suggesting their involvement in common biological processes. These analyses collectively demonstrated robust correlations between the differentially expressed genes and metabolites, providing insights into the integrated molecular mechanisms underlying the observed phenotypes.

#### 3.4.2. Enrichment Analysis of Signaling Pathways for Differential Genes and Metabolites

**Figure 9:**
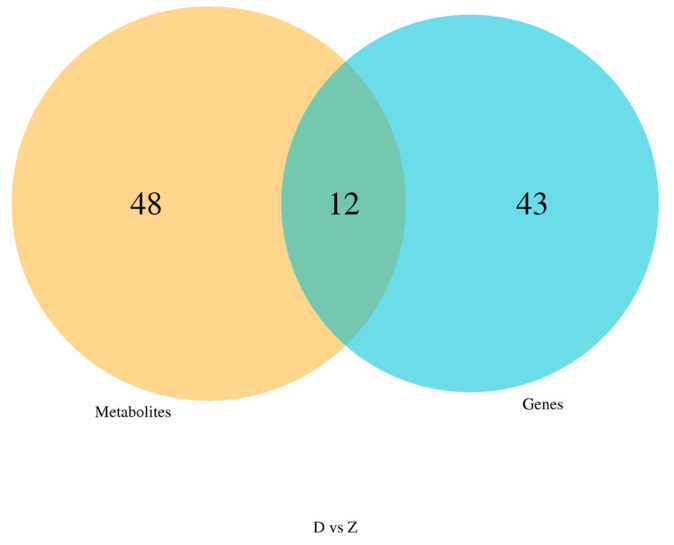
Venn Diagram of Pathways Enriched with Differential Genes and Metabolites.

**Figure 10:**
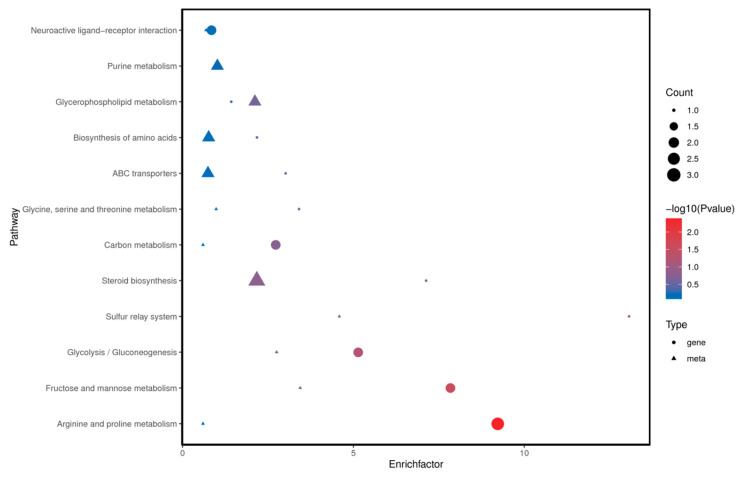
KEGG Enrichment Bubble Chart Including Pathways with Differential Genes/Metabolites.

As shown in Figure 9 and Figure 10, we identified a total of 12 signaling pathways that were enriched with both differential genes and metabolites. These primarily include Arginine and proline metabolism, Fructose and mannose metabolism, Steroid biosynthesis, Glycerophospholipid metabolism, and Glycolysis/Gluconeogenesis.

## 4. Discussion

Long-wave ultraviolet A (UVA) radiation has been shown to promote the synthesis of vitamin D3 (VD3) in animal skin. The primary function of VD3 is to enhance the absorption of calcium and phosphorus in the intestine, maintaining normal levels of these minerals in the body, which supports bone matrix calcification and improves animal growth performance [24]. In this experiment, we observed that at 45 days of age, the average body weight of groups subjected to 6 h of daily UVA exposure was significantly higher than that of the control group not exposed to UV *(p* < 0.05), and the average body weight increased as the exposure time increased. This result differs from the findings of Wang Haige [25], who discovered that 2 h of daily exposure to medium-wave ultraviolet radiation (UVB, with wavelengths ranging from 300–320 nm) could effectively improve the growth performance and skeletal development of broiler chickens; however, when the exposure time was increased to 4 h per day, there was no significant difference compared to the control group (*p* > 0.05). The reason for this discrepancy may be due to the different wavelengths of UV used in our study and Wang Haige’s study. Wang Haige used UVB, whereas this study utilized UVA (wavelength range 340–400 nm). There are differences in the biological effects of UVB and UVA, and different wavelengths can produce distinct physiological responses, thereby affecting growth performance and bone health.

Ultraviolet radiation is classified as a complete carcinogen, capable of inducing various adverse reactions such as erythema, tanning, photoaging, inflammation, and hyperpigmentation, and in severe cases, it can lead to skin cancer [26,27,28,29]. Ultraviolet radiation can also negatively impact the animal’s immune system, reducing the immune and antioxidant functions of broiler chickens [25,30]. Excessive UV exposure can also cause eye diseases, such as photokeratitis, cataracts, and macular photodamage [31,32,33]. In this experiment, we observed significant differences in mortality rates across the different UV exposure groups. Specifically, prolonged daily UVA exposure (6 h/day) was associated with detrimental effects on the health of Xichou Black-boned Chickens, leading to increased mortality. Conversely, shorter exposure durations of 1 h and 3 h did not significantly impact mortality, suggesting that moderate levels of UV exposure are likely safe for these chickens. These findings highlight the importance of carefully managing the duration of UV exposure to maximize the growth benefits while minimizing potential health risks.

Studies have shown that long-term exposure to UV radiation can promote melanin production in the skin through direct action on melanocytes and indirect stimulation of keratinocytes to release melanogenic factors [34]. Moreover, skin darkness can accurately reflect changes in melanin content in Black-boned chickens, and measuring the L value of the skin using a colorimeter is an effective method to assess skin darkness [19,35]. The results of this experiment show that the L values of the chest and leg skin in the 1 h, 3 h, and 6 h groups were lower than those of the control group (0 h), indicating that UV exposure can increase the darkness of Black-boned chickens and promote melanin production. Specifically, the L values of the chest and leg skin in the 1 h group were significantly lower than those in the 0 h and 6 h groups (*p* < 0.01).

Additionally, our study found that from 1 to 45 days of age, the L values of the chest and leg skin in Black-boned chickens gradually increased, and the skin darkness became lighter. This trend is consistent with previous research findings [36] and aligns with the results observed in Tengchong Snow Chickens, another local black-boned chicken breed in Yunnan Province [37]. Both Tengchong Snow Chickens and the Xiachou Black-boned Chickens studied here exhibit a similar age-related increase in skin L values, suggesting a common developmental pattern in melanin pigmentation among these breeds.

Studies have shown that the melanin content in blood can reflect the total amount of melanin in the body to some extent, and it mainly originates from melanin produced by skin melanocytes entering the bloodstream [38]. This experiment analyzed the melanin content data in the blood of different UV exposure time groups (0 h, 1 h, 3 h, 6 h) at 45 days of age. Although there was no significant difference between the four groups (*p* > 0.05), the melanin content in the 1 h group was slightly higher than in the other three groups, and the content in the 0 h group was the lowest. The trend in blood melanin content was: 1 h > 3 h > 6 h > 0 h, consistent with the previously observed changes in the L values of the chest and leg skin of Xichou Black-boned Chickens at 45 days of age. Combined with the measurement analysis of skin tissue L values and blood melanin content, the results confirm that 1 h of daily UV exposure is most effective in enhancing the darkness of Black-boned chickens.

Through the transcriptome analysis of the chest skin tissue samples from the 0 h and 1 h groups, we identified 22 differentially expressed genes that are primarily enriched in the mTOR and PPAR signaling pathways, which are associated with melanocytes and lipid metabolism. Studies have shown that unsaturated fatty acids (such as linoleic acid and alpha-linolenic acid) can promote the degradation of tyrosinase (*TYR*) through the ubiquitin-proteasome pathway, thereby inhibiting the production of melanin [39]. Peroxisome proliferator-activated receptors (PPARs), as nuclear hormone receptors activated by fatty acids and their derivatives, play an important role in the regulation of lipid metabolism and affect the differentiation of adipocytes [40]. In this study, differentially expressed genes such as *LPL* and *APOA1* were enriched in the PPAR signaling pathway. *LPL* is a key enzyme involved in the degradation of triglycerides into glycerol and free fatty acids, making it an important regulatory factor in lipid metabolism [41]. Additionally, *LPL* plays a significant role in fatty acid metabolism during the differentiation of preadipocytes into mature adipocytes [42,43], potentially affecting the melanin content in the skin of Xichou black-boned chickens by regulating the production and metabolism of unsaturated fatty acids.

The mechanistic target of rapamycin (mTOR) acts as an important intracellular sensor and is involved in the regulation of autophagy [44,45]. In the mTOR pathway, the gene *FZD3* was significantly upregulated in the 1 h group, and it is hypothesized that this enhances the inhibitory effect on autophagy, leading to an increase in the number of melanocytes and melanin content, thus deepening the melanin degree in the 1 h group.

Through metabolomic analysis of the chest skin tissue samples from the 0 h and 1 h groups, under the All mode, we ultimately significantly enriched two metabolic pathways closely related to melanin synthesis: the tryptophan metabolic pathway (containing the significantly differential metabolite L-tyrosine) and the biosynthetic pathway of unsaturated fatty acids (containing the significantly differential metabolite alpha-linolenic acid). Studies by Ando et al. have shown that alpha-linolenic acid can not only accelerate the renewal of the stratum corneum but also lighten pigmentation by inhibiting melanin production and promoting the shedding of melanin from the epidermis [46]. Additionally, alpha-linolenic acid can be converted into long-chain polyunsaturated fatty acids (LC-PUFAs), such as eicosapentaenoic acid (EPA) and docosahexaenoic acid (DHA) [47]. In this study, the metabolomic results showed that alpha-linolenic acid was significantly downregulated in the 1 h group, implying that its inhibitory effect on melanin synthesis is weaker, leading to an increase in melanin content and ultimately deepening the melanin degree in the 1 h group. Regarding the tryptophan metabolic pathway, research indicates that increased tryptophan levels inhibit the deposition of melanin in black-boned chickens, possibly by affecting the absorption, utilization, and metabolism of phenylalanine and tyrosine [48]; tyrosinase is a key enzyme in melanin synthesis, with L-tyrosine being catalyzed and oxidized to dopaquinone and eventually forming melanin [49,50]. The metabolomic results showed that L-tyrosine was significantly upregulated in the 1 h group, further leading to an increase in melanin content and a deepening of the melanin degree.

Through the correlation analysis between differential genes and metabolites, we found a significant positive correlation between *LPL* in the PPAR pathway and α-linolenic acid in the unsaturated fatty acid biosynthesis pathway. In the pectoral skin tissue of the 1 h irradiation group, both *LPL* and α-linolenic acid were significantly downregulated, leading to a weakened inhibitory effect of unsaturated fatty acids on melanin synthesis, resulting in increased melanin synthesis and ultimately causing the 1 h group to become darker.

Similarly, there was a significant positive correlation between *FZD3* in the mTOR pathway and L-tyrosine in the tryptophan metabolism pathway. In the pectoral skin tissue of the 1 h group, both *FZD3* and L-tyrosine were significantly upregulated, leading to increased melanin content and ultimately causing the 1 h irradiation group to become darker.

These findings suggest that the interplay between gene expression and metabolic changes can significantly influence the melanin synthesis process and ultimately the skin darkness of Xichou Black-boned Chickens.

## 5. Conclusions

This study investigated the effects of ultraviolet (UV) irradiation on body weight, skin melanin traits, and related molecular mechanisms in Xichou black-boned chickens through transcriptomic and metabolomic analyses. Preliminary observations indicate that under 47 μW/cm^2^ UV irradiation, the survival rate did not change significantly as long as the daily irradiation time did not exceed 3 h; however, when the irradiation time exceeded 3 h, the survival rate significantly decreased. Additionally, the L values of the chest and leg skin in the 1 h irradiation group were both lower than those in the control group, and the blood melanin content was highest in this group. These results suggest that one hour of daily UV irradiation may have a positive impact on body weight and skin melanin traits.

In terms of gene expression and metabolite changes, 22 differentially expressed genes and 365 differential metabolites were identified in the chest skin tissue between the 1 h irradiation group and the control group. Notably, genes such as *FZD3* were significantly enriched in the mTOR signaling pathway, while differential metabolites were significantly enriched in the tryptophan metabolic pathway related to melanin synthesis, particularly L-tyrosine. Comprehensive analysis revealed that *FZD3* in the mTOR pathway and L-tyrosine in the tryptophan metabolic pathway were significantly upregulated in the 1 h irradiation group, leading to an increase in melanin content and ultimately causing a deeper melanin degree in this group.

In summary, this study demonstrates that UV irradiation not only has potential positive effects on the body weight and skin melanin traits of Xichou black-boned chickens but also provides important clues for exploring the molecular mechanisms underlying these traits. Future research should further investigate the long-term effects of UV irradiation and provide more comprehensive data to validate the preliminary findings of this study.

## Figures and Tables

**Table 1:** Primer Information.

Gene Name	Primer Sequence (5′-3′)	Annealing Temperature (°C)
*LPL*	F:TGGACATTGGTGACCTGCTTATG	55
	R:TCGCCTGACTTCACTCTGACTCTC	
*HSP90AA1*	F:CGCGTTTGCTGATTCTGTGA	55
	R:GGTTGGTCCTGTGTTTGCAC	
*APOA1*	F:CTTGACCTGAAGCTGGCTGA	57
	R:CTCCTTCAGCTCCTCCTCCA	
*FZD3*	F:ATTCCTCCTCCCTGGGACTC	55
	R:GCTAAGCTGAGGTCTAGGCG	
*RXFP1*	F:CCCTTGGGCTACTTTCCCTG	60
	R:GCCCTCCAAGAGGGATTTGAG	

**Table 2:** Summary of Growth Performance and Survival Rate at 45 Days of Age.

Group	Average Body Weight (g)	Mortality (No. of Chickens)	Survivors (No. of Chickens)	Mortality Rate (%)
0 h	556.8 ± 51.0 ^b^	5	55	8.3
1 h	569.8 ± 72.5	5	55	8.3
3 h	572.6 ± 71.9	4	56	6.7
6 h	592.9 ± 74.6 ^a^	14	46	23.3

Data in the table are presented as mean ± standard deviation. Different lowercase letters (a, b) indicate significant differences between groups within the same column (*p* < 0.05). Groups labeled with the same letter or without a letter do not differ significantly (*p* > 0.05).

**Table 3:** Summary of Chi-Square Test Results for Mortality Rates.

Group	Chi-Square Contribution
0 h	0.571
1 h	0.571
3 h	1.286
6 h	7.000
Total	10.672

Statistical Results: Chi-Square Statistic (χ^2^): 10.672; *p*-value: 0.0136.

**Table 4:** Melanin Content in Blood at 45 Days of Age for Different UV Exposure Groups.

Group	Melanin Content (μg mL⁻^1^)
0 h	1.18 ± 0.09
1 h	1.21 ± 0.09
3 h	1.19 ± 0.08
6 h	1.19 ± 0.10

**Table 5:** Skin Hue (L Value) of Breast and Leg Skin at 22 and 45 Days of Age for Different UV Exposure Groups.

Group	22 Days of Age (Breast Skin L Value)	22 Days of Age (Leg Skin L Value)	45 Days of Age (Breast Skin L Value)	45 Days of Age (Leg Skin L Value)
0 h	44.3 ± 2.4 ^A^	46.4 ± 2.6 ^A^	57.1 ± 2.4 ^A^	56.7 ± 2.2 ^A^
1 h	42.5 ± 2.6 ^B^	40.7 ± 2.2 ^D^	47.0 ± 1.7 ^D^	47.4 ± 2.3 ^D^
3 h	42.9 ± 2.0 ^B^	42.8 ± 2.6 ^C^	53.2 ± 2.3 ^C^	53.8 ± 2.2 ^C^
6 h	44.2 ± 1.9 ^A^	44.0 ± 1.7 ^B^	55.7 ± 2.2 ^B^	55.2 ± 2.6 ^B^

Data in the table are presented as mean ± standard deviation. Different capital letters (A, B, C, D) indicate very significant differences between groups within the same column (*p* < 0.01). Groups labeled with the same letter or without a letter do not differ significantly (*p* > 0.05).

**Table 6:** Top 20 Significantly Enriched GO Terms.

GO ID	GO Term Description
GO:0004386	helicase activity
GO:0016251	RNA polymerase II general transcription initiation factor activity
GO:0000987	cis-regulatory region sequence-specific DNA binding
GO:0061659	ubiquitin-like protein ligase activity
GO:0140098	catalytic activity, acting on RNA
GO:0008134	transcription factor binding
GO:0051219	phosphoprotein binding
GO:0061630	ubiquitin protein ligase activity
GO:0008353	RNA polymerase II CTD heptapeptide repeat kinase activity
GO:0004467	long-chain fatty acid-CoA ligase activity
GO:0035198	miRNA binding
GO:0042974	retinoic acid receptor binding
GO:0001882	nucleoside binding
GO:0005347	ATP transmembrane transporter activity
GO:0016788	hydrolase activity, acting on ester bonds
GO:0005546	phosphatidylinositol-4,5-bisphosphate binding
GO:0042162	telomeric DNA binding
GO:0004693	cyclin-dependent protein serine/threonine kinase activity
GO:0042578	phosphoric ester hydrolase activity
GO:0070577	lysine-acetylated histone binding

**Table 7:** Distribution of the Top 20 Differential Metabolites by Category.

Metabolite Category	Number
Carboxylic acids and derivatives	44
Fatty Acyls	44
Organooxygen compounds	26
Steroids and steroid derivatives	25
Prenol lipids	24
Benzene and substituted derivatives	10
Quinolines and derivatives	8
Flavonoids	7
Glycerophospholipids	7
Sphingolipids	7
Diazines	6
Organonitrogen compounds	6
Coumarins and derivatives	4
Purine nucleotides	4
Unsaturated hydrocarbons	4
Macrolides and analogues	3
Pyrimidine nucleosides	3
Benzofurans	2
Benzoxazines	2
Diarylheptanoids	2
Total	238

## Data Availability

The data presented in this study is available in this article.
